# Histone deacetylases: Regulation of vascular homeostasis via endothelial cells and vascular smooth muscle cells and the role in vascular pathogenesis

**DOI:** 10.1016/j.gendis.2024.101216

**Published:** 2024-01-22

**Authors:** Hanyi Yang, Kai Guo, Peng Ding, Jiayi Ning, Yimeng Zhang, Yuanyong Wang, Zhaoyang Wang, Guanglin Liu, Changjian Shao, Minghong Pan, Zhiqiang Ma, Xiaolong Yan, Jing Han

**Affiliations:** a Department of Ophthalmology, Tangdu Hospital, The Air Force Military Medical University, Xi'an, Shaanxi 710038, China; b Xi'an Medical University, Xi'an, Shaanxi 710086, China; c Department of Thoracic Surgery, Shaanxi Provincial People's Hospital, The Third Affiliated Hospital of Xi'an Jiaotong University, Xi'an, Shaanxi 710068, China; d Department of Thoracic Surgery, Tangdu Hospital, The Air Force Military Medical University, Xi'an, Shaanxi 710038, China; e Department of Medical Oncology, Senior Department of Oncology, Chinese PLA General Hospital, The Fifth Medical Center, Beijing 100853, China

**Keywords:** Deacetylation, Endothelial cells, Histone deacetylase, Vascular disease, Vascular smooth muscle cells

## Abstract

Histone deacetylases (HDACs) are proteases that play a key role in chromosome structural modification and gene expression regulation, and the involvement of HDACs in cancer, the nervous system, and the metabolic and immune system has been well reviewed. Our understanding of the function of HDACs in the vascular system has recently progressed, and a significant variety of HDAC inhibitors have been shown to be effective in the treatment of vascular diseases. However, few reviews have focused on the role of HDACs in the vascular system. In this study, the role of HDACs in the regulation of the vascular system mainly involving endothelial cells and vascular smooth muscle cells was discussed based on recent updates, and the role of HDACs in different vascular pathogenesis was summarized as well. Furthermore, the therapeutic effects and prospects of HDAC inhibitors were also addressed in this review.

## Background

Histone deacetylases (HDACs) are generally divided into NAD^+^-dependent enzymes and Zn^2+^-dependent enzymes. The HDAC I, II, and IV subfamilies are Zn^2+^-dependent enzymes. The majority of NAD^+^-dependent enzymes are class III HDACs.^1^ The main function of HDACs is to maintain the equilibrium of histone acetylation in the basic unit nucleosome of chromosomes. One of the main regulating processes in histone octamer synthesis is its mediated deacetylation of lysine residues. Lysine residues carry positive charges, whereas DNA carries negative charges; thus, they attract each other. This attraction increases the density of chromatin structure and isolates promoters from transcription regulatory elements, hence limiting transcription. HDAC activity has been demonstrated to indirectly influence various posttranslational modifications, including phosphorylation, ubiquitination, and methylation, in addition to gene expression.^2^, ^3^, ^4^ These modifications are involved in various physiological and pathological processes, such as the cell cycle, angiogenesis, proliferation, differentiation, vascular permeability, and inflammation.^5^, ^6^, ^7^, ^8^ More significantly, increasing evidence suggests that HDAC dysfunction contributes to a variety of diseases, including cancers, neurodegenerative diseases, metabolic and immune diseases, inflammation, and fibrosis disease.^9^, ^10^, ^11^, ^12^, ^13^, ^14^, ^15^, ^16^ Thus, this paper describes the most recent research developments of HDACs in the vascular system, starting with two aspects of vascular physiology and pathology, and suggests several new future study options as well as novel hypotheses. Collectively, the data we present below will assist in the design of future studies and the identification of possible targets and techniques for vascular disease treatment in the future.

## Role of HDACs in vascular homeostasis

### Role in vascular cell homeostasis

The maintenance of vascular homeostasis is significant for normal physiological activities. Vascular homeostasis is largely determined by cell homeostasis.^17^ This section will elaborate on the regulation of HDACs on vascular cell homeostasis from the effects of HDACs on endothelial cells (ECs) and vascular smooth muscle cells (VSMCs).

## Role in ECs

### Role in endothelial differentiation

ECs are involved in the formation of arterial, venous, hemogenic, or lymphatic systems which make up the branching network of the vascular system.^18^ Therefore, ECs play a key role in vascular homeostasis. A series of studies have shown that HDACs can impact endothelial differentiation. HDAC3 has been shown to guide embryonic stem cells to EC differentiation through the p53-p21 pathway.^19^ Furthermore, the activation of HDAC3 is involved in shear stress and affected by vascular endothelial growth factor (VEGF), and siRNA-mediated knockdown of HDAC3 abolishes shear- and VEGF-induced EC marker gene expression.^19^ Moreover, valproic acid (VPA), a type of HDAC inhibitor (HDACi), reduced endothelial colony-forming cell differentiation and changed endothelial colony-forming cell morphology in an *in vitro* model.^20^ ECs have the potential to be used for revascularizing ischemic areas and engineering artificial blood vessels and tissues. The *in vitro* differentiation of ECs from pluripotent stem cells also provides a new opportunity for studying the molecular mechanisms that control the fate of ECs.^21^ These studies consistently suggest that HDACs are involved in induced EC differentiation, and provide insights into the use of stem cell therapy for vascular diseases in the future.

### Role in the migration and proliferation of ECs

HDACs are responsible for facilitating the migration and proliferation of ECs. One study demonstrated that HDAC1/2/3 mediated cyclin A up-regulation and p21 decrease in ECs, inducing the proliferation of ECs.^22^ Mottet and colleagues reported that scHDAC7 siRNA-transfected human umbilical vein endothelial cells showed up-regulated migration compared with HDAC7 siRNA-transfected human umbilical vein endothelial cells, as well as an increase in platelet-derived growth factor-B expression, suggesting that HDAC7 enhanced the migration of ECs by regulating the expression of platelet-derived growth factor-B.^23^ Another study showed that VEGF mediates the phosphorylation and nuclear export of HDAC7 via a protein kinase C/protein kinase D1 (PKD1) pathway, inducing EC migration and proliferation. Moreover, further study pointed out that the pharmacological inhibition of PKD could avoid HDAC7 phosphorylation, inhibiting the migration and proliferation of ECs.^24^ However, the side effect of PKD inhibition in clinical research has not been clarified, and whether PKD inhibition can act as a target in antiangiogenic therapy still needs to be confirmed by further experiments. Xu et al demonstrated that HDAC6 attenuated miR-155-5p expression, reducing Ras homolog enriched in brain expression, which enhanced microvascular EC proliferation.^25^ Most of the reports about the effect of HDACs on ECs to date were in pathological conditions. However, understanding the physiological regulation of HDACs on ECs is necessary for us to learn the mechanism of the pathological conditions. Thus, more research in this area is still needed.

## Role in VSMCs

VSMCs mainly exist in the medial layer of arteries^26^ and therefore mainly affect arteries rather than veins. They contribute to the regulation of vascular tone, vascular calcification, and vascular remolding.^27^^,^^28^ Therefore, VSMC regulation is significant for vascular homeostasis.

### Role in the differentiation of VSMCs

A study by Margariti et al showed that the spliced HDAC7 isoform stimulates smooth muscle actin and SM22 reporter gene expression in differentiated ES cells, inducing smooth muscle cell (SMC) differentiation from embryonic stem cells.^29^ The Notch ligand Jagged1 is a key driver of smooth muscle differentiation in the aortic arch arteries. Singh et al demonstrated that HDAC3 could decrease smooth muscle differentiation by regulating Jagged1.^30^ Zhang et al showed that HDAC6 inhibition thinned damaged neointima in the injured carotid artery *in vivo*, which is known to be formed largely by dedifferentiated SMCs. Meanwhile, HDAC6 inhibition promoted the differentiation of VSMCs *in vitro* by stimulating the myocardin-related transcription factor A/serum response factor pathway.^31^ In addition, SMC-restricted gene expression aids in the differentiation of SMCs. Dhagia and colleagues suggested that glucose-6-phosphate dehydrogenase mitigated the transcription of SMC-restricted genes through HDAC-dependent deacetylation, potentially aggravating the severity of vascular diseases associated with metabolic syndrome.^32^

### Role in the migration and proliferation of VSMCs

The sirtuin (SIRT) protein family belongs to class III HDACs and is widely studied in aging-related diseases.^33^ The study by Li and colleagues suggests that SIRT1 may be an attractive therapeutic target for the prevention of vascular diseases. SIRT1 repressed VSMC proliferation by inducing cell cycle arrest at the G1/S transition. In addition, up-regulated SIRT1 resulted in the significant inhibition of VSMC migration.^34^ Tubastatin A (TSA), an HDACi, reduces the expression level of thioredoxin 1, therefore up-regulating the proliferation and migration of VSMCs by enhancing the activation of Akt.^35^ HDACi butyrate has been shown to suppress VSMC proliferation through HDAC inhibition and phosphoinositide 3-kinase (PI3K)/Akt pathway network.^36^ In newborn pulmonary arterial SMCs, inhibition of class I and II HDACs by apicidin and HDACi VIII decreased proliferation and induced cell cycle arrest in the G1 phase.^37^ MGCD0103 is an HDACi that selectively inhibits class I HDAC-1/2/3. Cavasin et al demonstrated that the pulmonary arterial pressure of MGCD0103-treated rats was reduced by suppression of SMC proliferation.^38^ Jiang et al showed that tumor necrosis factor (TNF)-α and insulin-like growth factor 1 increased VSMC proliferation through elevation of DNA methyltransferase-1 expression in an HDAC2/10 dependent pathway.^39^ It is interesting to note that Kimura et al showed that SIRT7 deficiency attenuated the VSMC proliferation by regulating the expression of microRNA 290-295/CDK2, thus inhibiting neointimal formation after vascular injury.^40^ However, the study by Zheng et al suggested that SIRT7 inhibited VSMC proliferation through a Wnt/β-catenin-dependent pathway.^41^ This discrepancy may be due to the different animal species and study models used in these studies. Kimura et al investigated the effects and mechanisms of Sirt7 gene deletion on neointima formation after vascular injury using Sirt7 knockout mice (*Sirt7*^*−/−*^ mice) and wild-type mice as animal models and primary VSMCs as a cell model. Zheng et al established an atherosclerosis cell model using human VSMCs as a cell model and oxidized low-density lipoprotein as a stimulator. In summary, the two studies have opposite conclusions on the effects of the SIRT7 gene on VSMCs, which may be due to the differences in experimental models and mechanisms. This also indicates the complexity and importance of the SIRT7 gene in vascular biology, and more research is needed to reveal its exact functions and regulatory mechanisms.

## Role in the homeostasis of vascular components

The role of HDACs in vascular cell homeostasis has been discussed in the previous section. Vascular components are also involved in vascular homeostasis. Ribonuclease 1 (RNase1) is a vessel- and tissue-protective enzyme that regulates the vascular homeostasis of extracellular RNA. HDAC2 has been reported to regulate RNase1 expression by accumulating at the RNase1 promoter upon TNF-α stimulation.^42^ Further studies have shown that inhibition and knockout of p38 kinase- and HDAC2-associated kinase casein kinase 2 reversed the inflammatory inhibitory expression of RNase1.^43^ Moreover, siRNA knockdown of chromodomain helicase DNA binding proteins 3 and 4 of the nucleosome remodeling and deacetylase complex restored inflammatory-stimulated RNase1 inhibition.^43^ Together, the above studies revealed a TNF-α/p38/HDAC2-containing repressor complex/RNase1 axis to regulate RNase1 expression. Therefore, we suggested that combining inhibition of p38 and HDAC2-related complex might have the potential to protect vascular homeostasis.

## Role in vascular integrity

ECs also play an important role in the regulation and maintenance of vascular homeostasis and integrity. Several reports have suggested that HDACs negatively affect endothelial integrity. Heading date 3α (HD3α) is an HDAC3 splicing variant, and Zeng et al reported that overexpression of HD3α reprogrammed human aortic ECs into mesenchymal cells featuring an endothelial-to-mesenchymal transition phenotype, which was both PI3K/Akt- and transforming growth factor-beta 2-dependent.^44^ This study provides the first evidence that HDAC3 splicing is involved in the maintenance of endothelial integrity and enriches endothelial-to-mesenchymal transition-related pathways.

The endothelial glycocalyx is essential for maintaining normal endothelial function. Ali et al have verified the role of HDACs in mediating oxidative stress-induced up-regulation of matrix metalloproteinases in ECs, resulting in derangements of the endothelial glycocalyx. Inhibition of matrix metalloproteinases and HDACs reversed syndecan-1 and superoxide dismutase 3 shedding and maintaining endothelial glycocalyx integrity in oxidative stress-treated human adipose microvascular ECs.^45^ This study reveals that HDACs may affect vascular integrity by regulating the relationship between ECs and the extracellular matrix. These findings suggest that matrix metalloproteinases and HDACs are potential therapeutic targets for preserving the endothelial glycocalyx and preventing vascular complications in oxidative stress-related diseases. Further studies are needed to elucidate the molecular mechanisms and clinical implications of these interventions.

## Role of HDACs in vascular pathology

### Role in anti-angiogenesis

Angiogenesis, defined as the formation of new blood vessels from original vessels, plays a key role in cardiovascular disease and cancer.^46^^,^^47^ Epithelial-derived factor is an important anti-angiogenic and vascular protective factor. HDACi vorinostat (SAHA) had a cooperative effect with epithelial-derived factors at low concentrations and had an antagonistic effect at high concentrations by the NF-κB pathway.^48^ Kaluza et al found that knocking out or inhibiting the expression of HDAC9 could significantly inhibit the formation and collateral circulation of retinal vessels, thereby decreasing retinal vessel perfusion.^49^ Moreover, as a class I HDACi, largazole could significantly inhibit retinal vascular EC viability, proliferation, and the ability to form tube-like structures by inhibiting the expression of vascular endothelial growth factor receptor 2 (VEGFR2) and increasing the expression of p21. Although it has not been studied clinically, the potential role of largazole in modifying ocular angiogenesis through modulating VEGF-independent pathways is significant for patients who are less responsive to current anti-VEGF drugs.^50^

**Figure 1:**
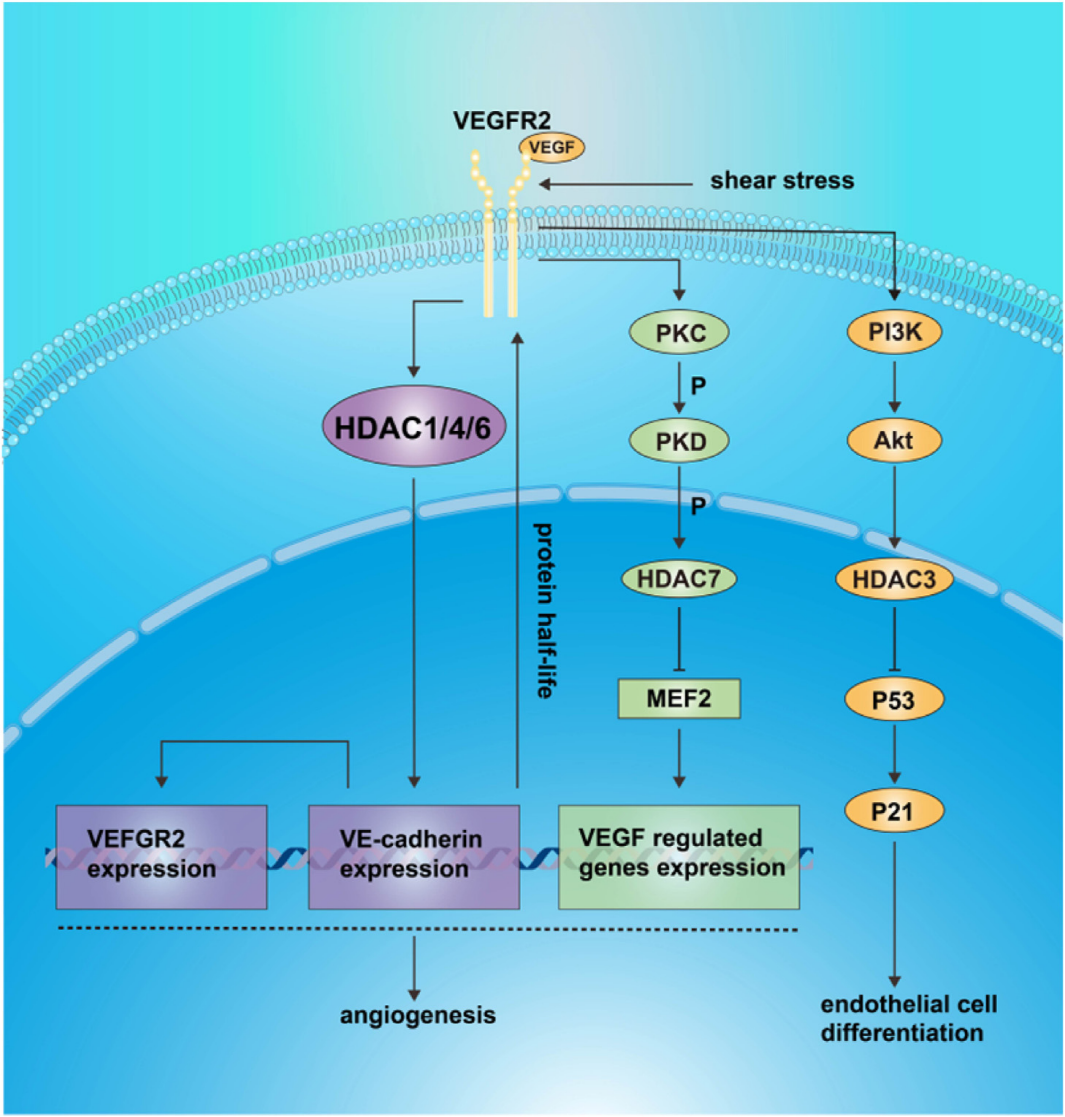
Schematic elucidation of the partial mechanism of VEGF involved in the regulation of HDACs. HDACs, histone deacetylases; MEF2, myocyte enhancer factor 2; VEGF, vascular endothelial growth factor.

Similarly, VPA has anti-angiogenic effects in the oxygen-induced retinopathy mouse model, which is associated with the inhibition of the VEGF-mTOR pathway.^51^ This study demonstrates that HDACi including VPA and SAHA suppress pathological retinal angiogenesis in mice with oxygen-induced retinopathy. It has been shown that the vascular pathology of oxygen-induced retinopathy is closely related to increased levels of VEGF^52^ and can be blocked by VEGFR inhibitors. This suggests that HDACi may be a new process to inhibit oxygen-induced retinopathy via a VEGF-related pathway.^53^ Additionally, HDACi TSA plays an antiangiogenic role via VE-cadherin-, HDAC1-, and HDAC4/5/6-mediated suppression of VEGFR-2 expression.^54^ Taken together, the above results indicate that HDACs are closely involved in angiogenic regulation, and HDACi may be a switch that regulates the balance of angiogenesis. Additionally, HDAC-mediated angiogenesis is closely related to the VEGF pathways and provides a new therapeutic target for retinal vascular diseases. Because VEGF plays an important role in HDAC regulation, we summarized the VEGF-related HDAC signaling pathways in this review (Fig. 1).

## Role in vascular inflammation

### Association with inflammatory cytokines

HDACs modulate vascular inflammation through various mechanisms. Among them, inflammatory cytokines are significantly involved in the process. Entinostat (MS275), a specific inhibitor of class I HDACs, could decrease proinflammatory cytokines such as TNF-α, interleukin (IL)-1β, and monocyte chemoattractant protein 1, as well as some adhesion molecules.^55^ Bedenbender et al found that HDAC2 inhibition abolished the TNF-α- or IL-1β-mediated effect on the mRNA and chromatin levels of RNase1 by increasing histone acetylation at the RNASE1 promoter site in ECs.^42^ On the other hand, as an HDAC8 selective inhibitor, PCI34051 could reduce the expression of inflammatory molecules, vascular cell adhesion molecule-1, and intercellular cell adhesion molecule-1 in the aortas of angiotensin (Ang) II-infused mice, modulating the activity of inflammatory cytokines.^56^ Research showed that HDAC3 inhibition by a specific inhibitor or genetic knockdown reduced the expression of inflammatory cytokines, such as TNF-α, IL-6, and monocyte chemoattractant protein-1, in the aortic endothelium.^57^ Moreover, Yang et al showed that HDAC4 induced the expression of vascular cell adhesion molecule-1 and IL-6 by activating autophagy in primary endothelial cells isolated from mice and rats.^58^ These studies suggest that HDACs play a crucial role in regulating vascular inflammation by modulating the expression of various cytokines and adhesion molecules.

### Association with inflammatory-related proteins

In addition to cytokines, other inflammatory-related proteins also play a significant role in inflammation. HDACs can regulate the expression of inflammatory proteins in the course of atherosclerosis. Arginase 2 (Arg2) is a critical regulator in atherosclerosis progression that controls vascular inflammation. HDAC2 overexpression in human aortic ECs suppressed Arg2 expression, and knockdown of HDAC2 by siRNA enhanced Arg2 expression. Moreover, HDAC2 overexpression attenuates oxidized low-density lipoprotein-mediated activation in human aortic ECs.^59^ Reactive oxygen species (ROS) are a class of highly reactive oxygen radicals that can cause oxidative stress and cell inflammation.^60^ NADPH oxidases (Nox) are a class of NADPH-dependent oxidases that can promote the production of ROS.^61^ Manea et al found that treatment of *ApoE*^*−/−*^ mice with the Pan-HDACi SAHA significantly reduced the extent of atherosclerotic lesions and the aortic expression of Nox subtypes, NADPH-stimulated ROS production, oxidative stress, and proinflammatory markers.^62^ Additionally, HDAC11 promoted the acetylation levels of the ETS-related gene, inducing pyroptosis by NOD-like receptor thermal protein domain associated protein 3/caspase-1/gasdermin D and caspase-3/gasdermin E pathways in ECs.^63^

### Association with inflammatory cells

Apart from the intervention factors described above, inflammatory cells, such as macrophages, are key candidates responsible for vascular inflammation and atherosclerosis. Compared with wild-type mice, the atherosclerosis in HDAC3 deletion mice had a more stable, less inflammatory profile indicating a beneficial macrophage phenotype.^64^ Additionally, SAHA has been shown to induce pharmacological inhibition of HDACs, further repressing the gene expression of Nox subtypes in cultured pro-inflammatory macrophages.^62^ On the other hand, SIRT1 induced deacetylation of RelA/p65-NF-kB, suppressing the expression of Lox-1, a scavenger receptor for oxidized low-density lipoprotein, thereby preventing macrophage foam cell formation.^65^ The above studies indicate that HDACi and SIRT1 both repress inflammation and may have the potential for vascular protection.

### Association with vascular plaque-related inflammation

In atherosclerosis, inflammation mediates the formation and stability of vascular plaques through a variety of signaling molecules and pathways.^66^ Plaque formation is a potentially dangerous chronic pathologic process that may suddenly cause life-threatening coronary thrombosis.^67^ HDACs play a pathogenic role in vascular plaque formation. *In vivo*, atherosclerosis-prone mice with endothelial-specific HDAC9 knockout showed reduced endothelial-to-mesenchymal transition, significantly reduced plaque area, and a more favorable plaque phenotype, with reduced plaque lipid content and increased fibrous cap thickness.^68^ Moreover, HDAC9 has been shown in hyperlipidemic mice to bind to IKKα/β, resulting in their deacetylation and subsequent activation, driving inflammatory responses in both macrophages and ECs. The HDAC9 selective inhibitor TMP195 reduced lesion formation by decreasing endothelial activation and leukocyte recruitment along with limiting proinflammatory responses in macrophages. TMP195 also decreased the progression of established lesions, inhibited the infiltration of inflammatory cells, and enhanced plaque stability in advanced lesions by reducing features of plaque vulnerability.^69^ In addition, through partial SIRT1 deletion in atherosclerotic mice, Stein et al demonstrated that SIRT1 reduced macrophage foam cell formation by down-regulating the uptake of oxidized low-density lipoprotein.^70^

From the above studies on vascular inflammation, although some specific and pan-HDAC inhibitors have shown effective inhibition of vascular inflammation, further studies are needed to evaluate their pharmacological and pharmacokinetic profiles.

### Association with perivascular fibrosis

Inflammation is an important trigger of fibrosis, and fibrosis is one of the outcomes of inflammation.^71^ As a major therapeutic target for cardiovascular diseases, perivascular fibrosis is gaining attention for deeper exploration.^72^ Several pieces of research are collected to expound on the role of HDACs in perivascular fibrosis as follows. Guo and colleagues found that endothelial-specific loss of SIRT6 aggravated perivascular fibrosis in hypertensive mice, suggesting that SIRT6 may have an anti-perivascular fibrosis effect.^73^ In addition, the study by Tang et al reported that loss of SIRT2 can significantly increase myocardial fibrosis by attenuating AMP-activated protein kinase activation in aging mice and Ang II-infused mice.^74^ Similarly, HDAC3 inhibition as well as both VPA and RGFP-966, an HDAC3-specific inhibitor, significantly reduced the levels of the profibrotic cytokines plasminogen activator inhibitor-1 and connective tissue growth factor at 6 mo in OVE26 mice.^75^ Moreover, Overexpression of HDAC1 contributed to accelerated conjunctival fibrosis after trabeculectomy in a rat model. Correspondingly, TSA has been shown to ameliorate conjunctival fibrosis in bleb vascularity and leukocyte infiltration and decrease the expression of α-smooth muscle actin and TGF-β1.^76^ According to another research, HDACi largazole increased the acetylation of histones H3 and H4, further reducing the expression of collagen I, α-smooth muscle actin, and tissue inhibitor of metalloproteinase-1, which plays an antifibrotic role.^77^ According to the above studies, SIRT2/6 and HDACi both exert antifibrotic effects, which may prove to be attractive targets for vascular fibrosis therapy.

### Association with vascular calcification

Vascular calcification is thought to be associated with drivers of inflammation, oxidative stress, aging, *etc*.^28^ Calcification was regarded as a passive process in response to metabolic diseases and vascular diseases before, while now it is defined as a highly-regulated consequence.^78^ It has been found that murine double minute 2 (MDM2; HDAC-E3 ligase)-induced ubiquitination of HDAC1 is positively involved in the development of vascular calcification. Thus, regulation of HDAC activity and its protein stability by MDM2 may be a therapeutic target of vascular calcification.^78^^,^^79^ In addition, the HDACi TSA has been reported to promote the mineralization of human aortic SMCs by increasing the expression of alkaline phosphatase, inducing vascular calcification.^80^ It has been shown that butyrate can accelerate vascular calcification through the dual effects of HDAC inhibition and NF-κB activation.^81^ However, the specific process by which HDACs regulate vascular calcification has not been clarified. Further studies about the HDAC regulatory mechanisms in vascular calcification need to be carried out.

## Role in vascular permeability damage

Vascular permeability damage is involved in various diseases, such as acute lung injury and cerebral ischemia/reperfusion injury.^82^, ^83^, ^84^ Emerging reports have shown that HDACs can increase vascular permeability. A study by Zhao et al showed that RGFP966, the HDAC3 selective inhibitor, significantly attenuated the increase in trans-endothelial cell permeability by peroxisome proliferator-activated receptor gamma protein acetylation/activation.^85^ In addition, HDACi could mitigate the activation of heat shock protein 90 by inhibiting the chaperone function of HDACs, decreasing trans-endothelial hyperpermeability.^86^ Furthermore, a series of experiments have shown that HDACi TMP269 and TSA could down-regulate endothelial permeability via an Arg kinase binding protein 2-dependent pathway in lipopolysaccharide-treated ECs.^87^ Shi et al found that HDAC9 was involved in oxygen-glucose deprivation-induced EC permeability dysfunction in brain microvessel ECs.^88^ Taken together, inhibition of HDACs may attenuate increased vascular permeability in pathological conditions.

## Role in vascular tone dysfunction

**Figure 2:**
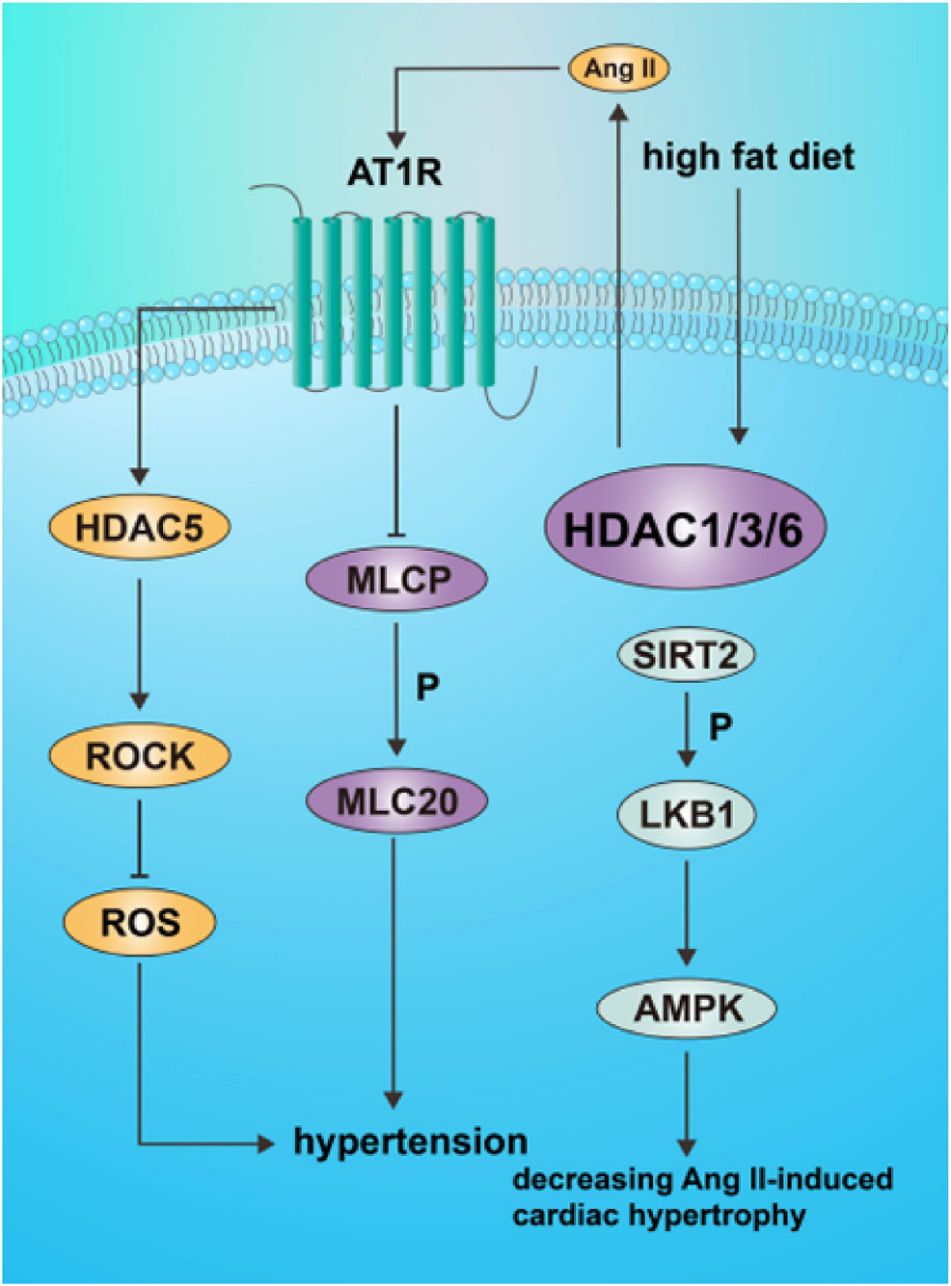
Schematic elucidation of the partial mechanism of Ang II involved in the regulation of HDACs. Ang II, angiotensin II; HDACs, histone deacetylases; ROCK, Rho-associated coiled-coil containing protein kinase.

Vascular tone is primarily modulated by mechanisms that regulate VSMCs, such as the renin-angiotensin-aldosterone system, sympathetic nervous system, and oxidative stress.^89^ Vascular tone dysfunction leads to abnormal relaxation or tension of blood vessels. There are two major ways in which HDACs modulate the process of vasomotion. One is to promote vasoconstriction and the other is to regulate vascular constriction mainly mediated by Ang II. A study by Bai et al showed that knockdown of HDAC5 reduced vasoconstriction and oxidative stress in an Ang II-induced hypertension model.^90^ Yoon et al demonstrated that the HDACi CG200745 led to the inhibition of increasing serum Ang II and vasoconstriction by inhibiting the high-fat diet/HDAC/Ang II/deactivated myosin light chain phosphatase/myosin light chain_20_ phosphorylation–vasoconstrictor axis.^91^ It is important to note that the inhibition of CG200745 on HDACs occurred only in the high-fat diet group, not in the normal diet group, suggesting that this inhibition blocks high-fat diet-induced increased expression of HDACs. More interestingly, CG200745 only down-regulated the expression of HDAC1/3/6, not HDAC2, which may be due to HDAC2 not having the function of nuclear export. Therefore, we hypothesize that a high-fat diet may regulate the expression of HDACs in the cytoplasm. Furthermore, as an HDAC8 selective inhibitor, PCI34051 could make vascular relaxation more dependent on vascular ECs in an Ang II-regulated mouse model, and this effect could be blocked by a nitric oxide synthase inhibitor. PCI34051 could also increase nitric oxide production in human umbilical vein endothelial cells, which was reduced by the nitric oxide synthase inhibitor.^56^ Homoplastically, HDACi MS275 could decrease the components of the renin-angiotensin system and enhance the vascular relaxation of rat aortic rings by the nitric oxide pathway.^55^ The above studies suggest that Ang II is directly or indirectly involved in the regulation of vasoconstriction by HDACs. Ang II-related signaling pathways have been summarized (Fig. 2). Consequently, Ang II inhibition by HDACi may provide a potential therapeutic method for hypertension treatment.

On the other hand, HDACs impact vasoconstriction by other mechanisms instead of Ang II. The study by Choi et al showed that HDAC4 and HDAC5 selective inhibitor LMK235 increased nitric oxide production in human umbilical vein endothelial cells and inhibited the increase in aortic wall thickness in animal hypertensive models, thus reducing vascular hyperplasia or vasoconstriction.^92^ Another study showed that the HDACi SAHA and TSA could inhibit L-type calcium channels and another mechanism-independent L-type calcium channel, leading to acute vasodilation.^93^ In addition, prostaglandin E2 reduced smooth muscle tone and changed proliferation and inflammatory activity. Fork et al pointed out that inhibition of HDACs reduced vascular prostaglandin E2 levels in the murine vasculature and human VSMCs.^94^ In short, HDACs may induce vasoconstriction via various mechanisms, including Ang II, prostaglandin E2, and calcium channels. Considering that Ang II-dependent vascular constriction is a long-term mechanism of blood pressure regulation, while prostaglandin E2 and calcium channels and other non-Ang II-dependent pathways are acute modulators, manipulating HDAC or HDACi to regulate Ang II may have potential to improve the long-term prognosis of vascular diseases, and studying their effects on non-Ang II-dependent pathways of vascular constriction may help avoid the adverse effects of acute vascular dysregulation associated with the use of HDAC-related drugs.

## Role in pathologic vascular remodeling

A great deal of studies have confirmed that HDACs are involved in vascular remodeling by regulating ECs and VSMCs, which may accelerate lumen narrowing. Studies on arterial remodeling in mice with Ang II hypertension showed that class II HDACi could negatively regulate VSMC hypertrophy.^95^ Moreover, knocking down HDAC5, Rho-associated coiled-coil containing protein kinase 1, and Rho-associated coiled-coil containing protein kinase 2 induced a decrease in VSMC hypertrophy in the aortas of Ang II-treated HDAC5-knockout mice.^90^ Li et al demonstrated that HDAC1 inhibited miR-34a level and subsequently up-regulated the ratio of matrix metalloproteinase 9/tissue inhibitor of metalloproteinase 1 and the ratio of matrix metalloproteinase 2/tissue inhibitor of metalloproteinase 2, inducing pulmonary arterial remodeling in monocrotaline-induced pulmonary arterial hypertension rats.^96^ ROS plays a role in the development and progression of maladaptive myocardial remodeling through various cellular events, such as the activation of hypertrophy signaling kinases, transcription factors, apoptosis, and cardiac fibroblast proliferation.^97^ Nox expression induces ROS production and oxidative stress, resulting in downstream endothelial dysregulation, vascular inflammation, and vascular wall thickening are important pathological mechanisms of diabetes.^98^ HDACs mediate Nox up-regulation in diabetes which induces ROS generation. HDAC inhibition reduced the production of vascular ROS in experimental diabetes by negative regulation of Nox expression.^99^ Another study showed that MGCD0103 attenuates aortic remodeling in rats with TAC-induced pressure overload.^100^ HDAC6 inhibition blocked the cigarette smoke-induced pulmonary arterial wall thickening, right ventricular systolic pressure, and right ventricular hypertrophy by the extracellular signal-regulated kinase pathway in chronic obstructive pulmonary disease.^101^ In addition, Ang II and mechanical stretch stimulated microtubule redistribution and deacetylation via SIRT2 in ECs, suggesting the emerging role of SIRT2 in hypertension-induced vascular remodeling.^102^ Although there have been many reports of HDACs regulating vascular remodeling, the molecular mechanisms are still unclear, such as in SMCs^103^ and extracellular matrix.^104^ More studies are needed to be carried out in this field.

## Role in tumor anti-vascular therapy

Goehringer et al reported that epidermal growth factor (EGFR) and HDAC2/6 inhibition showed pronounced anti-proliferative, apoptosis-inducing, and anti-angiogenic effects in solid tumor cell models.^105^ In addition, combinational inhibition of HDAC1/4 and phospholipase D1 decreased invasion, angiogenesis, colony-forming capacity, and self-renewal capacity than those of either treatment in glioblastoma.^106^ Based on VEGF inhibitor pazopanib and HDACi, Zang et al developed two dual-targeting inhibitors, and they both exhibited anti-angiogenesis and anti-proliferation effects.^6^ Moreover, a dual-targeting inhibitor of tubulin and class IIa HDACs showed anti-proliferative and anti-angiogenic effects in various tumor cell lines.^107^ Another study reported that a 4,5,6,7-tetrahydrobenzothiazole synthesized hydroxamic acid-based HDACi showed anti-migration and anti-angiogenesis activities in tumor cells.^108^ Similarly, dual targeting inhibitors of poly(ADP-ribose)polymerase-1 and HDAC6 exerted anti-proliferation activity and significant anti-migration and anti-angiogenesis effects.^109^ It is noteworthy to mention that the combined inhibition of HDACs and other targets has markable anti-tumor and anti-vascular effects. Moreover, drug combinations also have the benefit of reducing drug resistance. These findings enlighten us to dig deeper into combination drug therapy and synthetic HDAC-related multi-target drugs for tumor anti-vascular therapy in the future.

## Role in blood–brain barrier injury

The blood–brain barrier (BBB) is defined as a continuous endothelial membrane within brain microvessels that has sealed cell-to-cell contacts^110^ and controls the influx and efflux of biological substances.^111^ As well known, BBB injury is involved in a variety of neuropathies, including Alzheimer's disease. Wang et al demonstrated that VPA could inhibit matrix metalloproteinase 9, tight junction proteins, and nuclear translocation of NF-κB, attenuating BBB injury in transient middle cerebral artery occlusion rats.^112^ HDAC3 inhibition could oppose oxygen glucose deprivation and reoxygenation-induced increase of transendothelial cell permeability by inducing peroxisome proliferator-activated receptor gamma protein acetylation/activation. Another study reported that HDAC3 inhibition also improved BBB permeability in type 2 diabetes mellitus by Nrf2 activation.^113^ Thus, HDAC3 inhibition might exhibit therapeutic effects on BBB integrity injury.^85^ Soares Romeiro et al showed that cashew nut shell liquid derivatives could modulate glial cell-induced inflammation and revert the pro-inflammatory phenotype through an HDACi-like effect in Alzheimer's disease.^114^ The combination of plant extraction technology and HDACi is an innovation in this study, which suggests a new treatment strategy for Alzheimer's disease. However, the specific mechanisms of cashew nut shell liquid are unclear and need more investigation. In short, HDACi plays a crucial role in BBB injury through various mechanisms and may become a new therapeutic strategy for BBB-related diseases.

## Discussion

HDACs play a significant role in the epigenetic regulation of a variety of disorders, such as cardiovascular diseases^115^ and cancer.^116^ Numerous studies reviewed in this work demonstrate that HDACs can regulate the expression of angiogenesis-, fibrosis-, inflammation-, and Ang-associated genes and further control the epigenesis of related vascular diseases by modulating deacetylation or through other mechanisms. HDAC3 acts as a deacetylase in the TGFβ2-mediated epithelial-to-mesenchymal transition,^44^ and it also enhances the phosphorylation of Akt to maintain endothelial integrity under disturbed blood flow conditions.^117^ Moreover, studies have found that HDACi apicidin can regulate neonatal pulmonary hypertension by modulating methylation levels.^118^ Therefore, exploring the non-acetylation epigenetic functions of HDACs may be a new direction for investigating the mechanisms and therapeutic methods of HDAC-related vascular diseases. Downstream pathways of HDACs in vascular regulation have been extensively studied, such as VEGF-, Ang II-, and cell cycle-related pathways. However, few upstream regulators of HDACs in vessels have been reported. The study of upstream regulatory factors is crucial in further understanding the mechanisms of HDACs in various physiologic and pathologic conditions, which is not only important for the development of upstream targeted drugs, but also provides more information for avoiding drug side effects. For example, MDM2 regulates HDAC1 activity by ubiquitination, inducing vascular calcification.^79^ This finding indicates that posttranslational modifications of HDAC may play a role in vascular calcification, and this may become a promising direction in new drug development. Moreover, there are few studies on the role of class III and class IV HDACs in vascular and fibrotic diseases. More research on different classes of HDACs will give us new insights into understanding the function of HDACs and the pathology of vascular diseases.

When we discuss the role of HDACs in vascular physiology and pathology, we refer to a large number of *in vitro* and *in vivo* experiments. *In vitro* experiments mainly study the effects of HDACs on vascular cell phenotypes such as proliferation, migration, and differentiation,^24^^,^^30^ which provide us with insights into pathogenesis and molecular regulation from a microscopic perspective. *In vivo* experiments further verify the role of HDACs and explore the efficacy of HDACi in disease models, laying the foundation for the further clinical translation of the research results. The current *in vitro* experiments have all proven that HDACs play an important role in the atherosclerosis model, oxygen-induced retinopathy mouse model, hypertension model, high-fat diet model, *etc*.,^51^^,^^57^^,^^90^^,^^91^ which provide valuable experience for future drug development and efficacy verification in the HDAC field.

The therapeutic role of HDACi has been studied extensively^115^^,^^116^ and five HDACi have been approved for the treatment of different cancer types. However, cancer treated with HDACi alone always develops resistance and relapses due to tumor heterogeneity^119^ and microenvironment.^120^ In recent years, dual targeting HDACi has drawn wide attention, and several new drugs have been designed. Dual targeting HDACi has shown a high therapeutic potential for vascular diseases, such as cardiac hypertrophy, inflammatory, cardiovascular, neuromuscular illnesses, and cancer.^121^ One of the reasons for the good efficacy of dual targeting HDACi is their low drug resistance, which is essential because inhibition of another target prevents activation or reactivation of other HDAC-related pathways. For example, a new drug developed by Dong et al is based on an EGFR inhibitor and an HDACi.^122^ EGFR is a receptor in the HDAC pathway that regulates angiogenesis.^50^ The resistance of the EGFR inhibitor increases because EGFR reactivation is blocked by EGFR inhibitor activity. The combination of HDACi thus provides a supplement way and exhibits a better therapeutic effect. Moreover, the synthesis of dual-targeting HDACi from highly selective HDACi is also a potential strategy. Dual targeting HDACi synthesized by Wang et al showed highly selective inhibition of HDAC7, which depended on the selection of class IIa HDAC selective inhibitors as substrates.^107^ Highly selective HDAC inhibition may result in fewer systemic side effects.^114^^,^^123^^,^^124^ In addition, this inheritance of the original substrate inhibition properties makes the dual-targeting HDACi design more flexible. Despite the many benefits of dual targeting HDACi, their pharmacokinetics such as permeability are not ideal because their zinc-binding groups are composed of hydroxamates..^125^ Altering the low permeability of drugs by changing the zinc-binding groups might be a research direction for dual-targeting HDACi synthesis. Despite the difficulties, four dual-targeting HDACi have been put into clinical trials with satisfactory results.^126^ Additionally, HDACi combining other targets to modulate immune-related molecules in cancer also has shown a rapid development.^127^ Therefore, dual targeting HDACi is still a promising direction for HDACi development and will be the focus of future HDACi research.

Although a large number of studies have reported the effect of HDAC/HDACi on vascular diseases, some gaps warrant further investigations. Although dual-target HDACi is rapidly developing in cancer therapy, they have not been studied in cardiovascular disease. Mathew et al have shown that butyrate induces VSMC growth by dual targets of HDAC and PI3K/Akt pathway, and further regulates vascular remodeling.^36^ The discovery of these two targets may enlighten us in the future study of dual targeting HDACi in cardiovascular disease. Hyperuricemia plays a role in the pathogenesis of cardiovascular diseases,^128^ and uric acid is an independent risk factor for atherosclerosis and regulates VSMC proliferation, lipids, inflammation, *etc*., which subsequently induces cardiovascular disease.^129^ However, there are no studies on the regulation of uric acid metabolism by HDAC/HDACi, which may become a future research direction. It is worth noting that HDACi is beneficial in retinal diseases as well. Recently, HDACi has been shown to inhibit retinal neovascularization in cellular and animal models through VEGF-/VEGFR-related pathway.^50^^,^^51^ Interestingly, G570, a dual targeting HDACi that inhibits HDAC6/heat shock protein 90, has been shown to inhibit retinal neovascularization and blue light-induced cell migration in age-related macular degeneration.^130^ This finding opens up the possibility of dual targeting HDACi in retinal diseases, which may become the focus of future research. However, there are still many difficulties in the development of HDACi in retinal disease treatment. At present, almost all HDACi inhibit retinal neovascularization through VEGF-/VEGFR-related pathways,^51^, ^52^, ^53^^,^^130^ which may lead to drug resistance in long-term use. Thus, the development of new non-VEGF-dependent pathways, combined drugs, and dual-targeting drugs is the direction of future development. In addition, drug design for better penetration through the cornea is also a challenge in ocular disease treatment. Since many vascular phenotypes in tumor diseases are similar to those in cardiovascular and retinal diseases, and the effect of HDACi on tumors has been extensively studied, further research to confirm the role of these mechanisms in cardiovascular and retinal diseases may contribute to the development of novel therapeutic strategies.

## Conclusion

**Figure 3:**
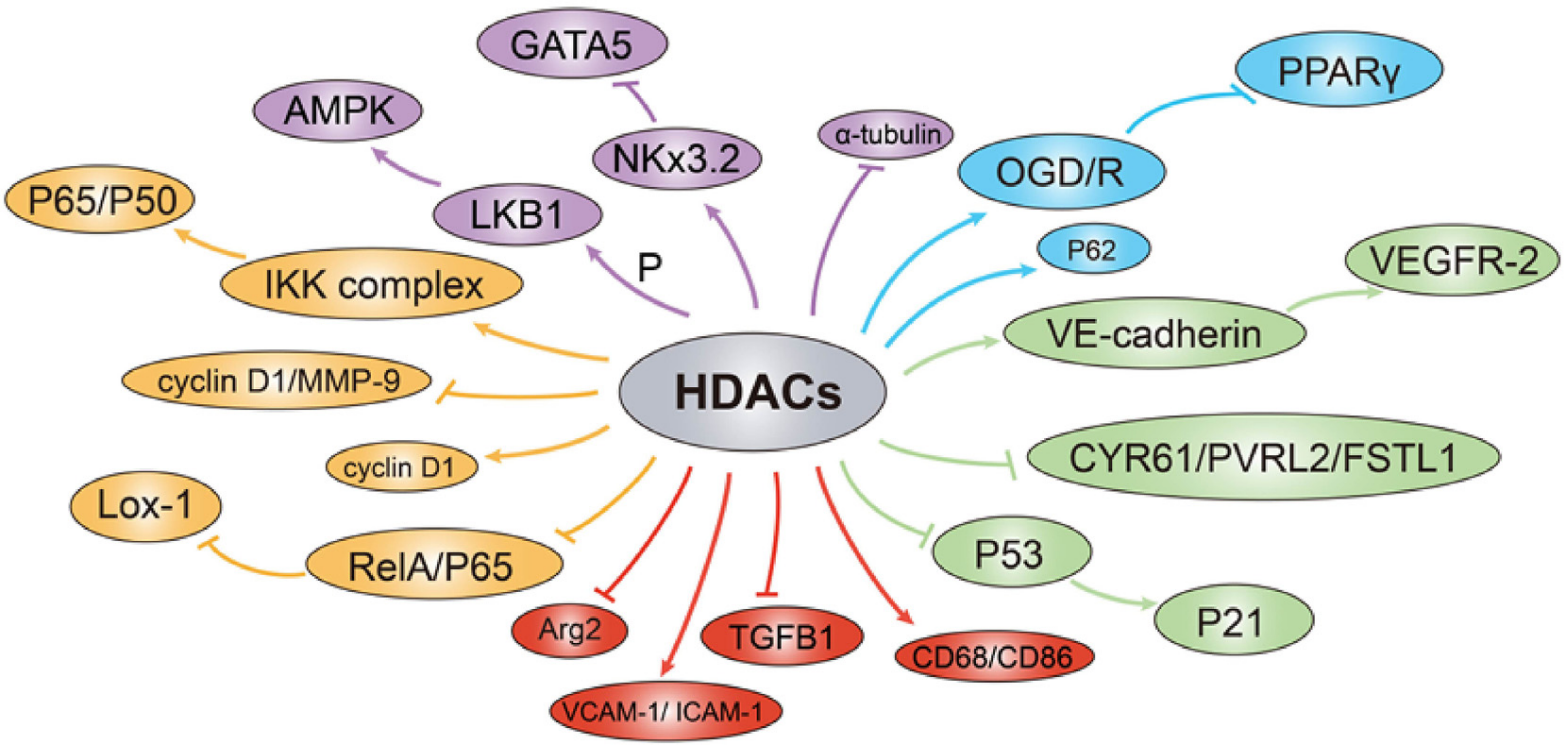
Fibrosis-related (shown by purple), permeability-related (shown by sky blue), VEGF-related (shown by green), inflammation-related (shown by red), and cell cycle-related (shown by orange) HDAC signaling pathways according to different functions. CYR61, cysteine-rich 61; FSTL1, Follistatin-like 1; HDAC, histone deacetylase; VEGF, vascular endothelial growth factor.

**Figure 4:**
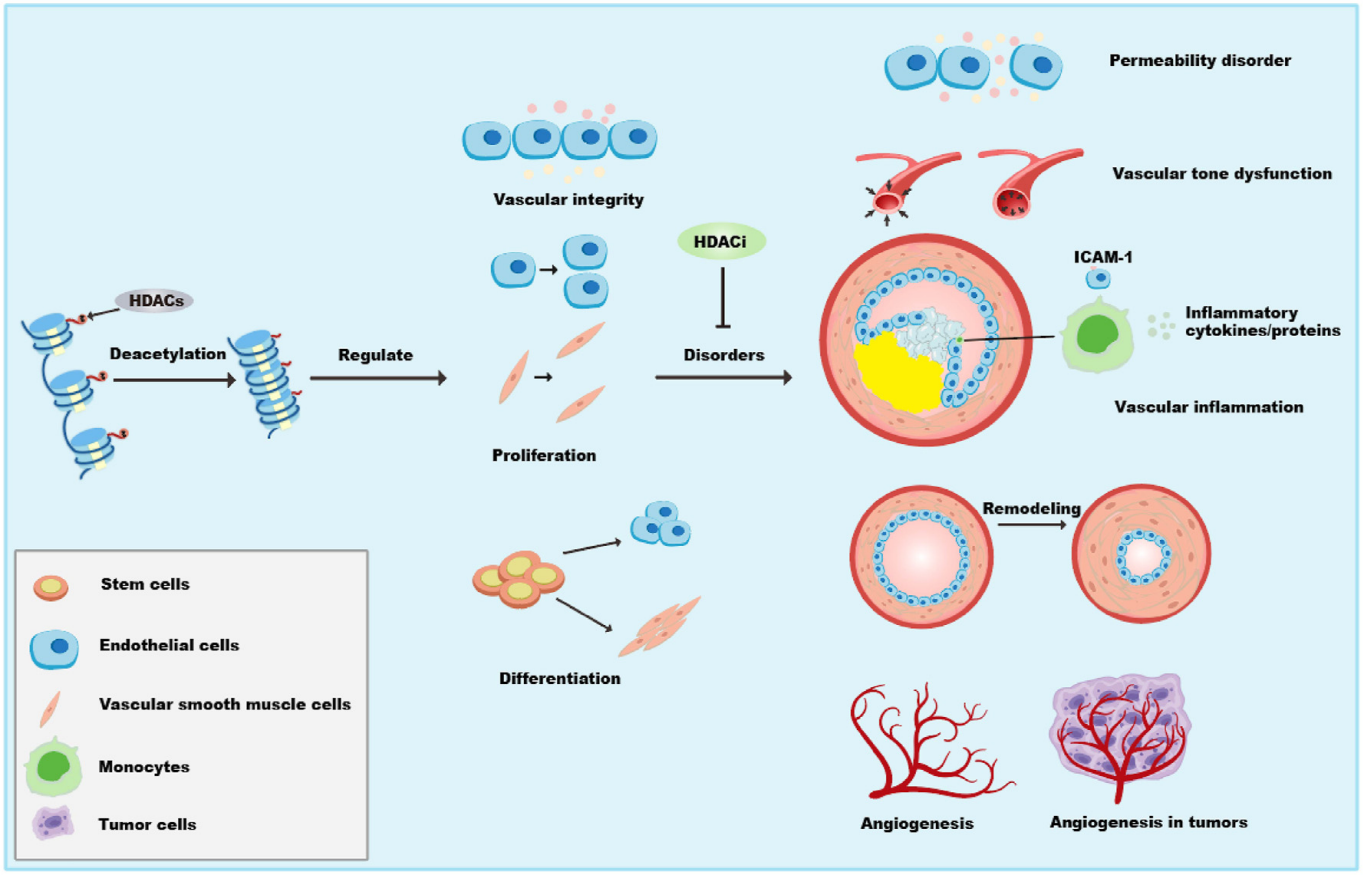
The schematic diagram of how HDACs regulate angiogenesis, vascular inflammation, vascular tone dysfunction, permeability disorder, and vascular remodeling. HDACs, histone deacetylases.

Recent progress in understanding the role of HDACs in vascular homeostasis and pathology is developing at a staggering rate (Table 1), thereby providing new insights into our understanding of the essential function of HDACs. Here, we summarized the regulatory role and mechanisms of HDACs in light of the present knowledge, such as vascular cells, inflammation, and tumor growth (Figure 3, Figure 4). Therefore, HDACs have profound potential to become therapeutic targets in vascular-related diseases. Even though HDACi have been shown to attenuate the pathological epigenesis in various disease models, more effective and less toxic drugs need to be developed, and more clinical trials for HDACi are needed.

**Table 1:** The role of HDACs in vascular physiology and pathology.

HDAC type	Category	Diseases or phenotypes	Upstream regulators	Functions	Related molecules/pathways	HDACi type	Reference
HDAC1	Class I			HDAC inhibition plays an anti-angiogenic role	VEGF/VE-cadherin	TSA	54
		Vascular calcification	MDM2	Ubiquitination of HDAC1 mediates vascular calcification	MDM2		79
HDAC2	Class I		Proinflammatory cytokines	Inhibiting the EC transformation	RNase1	MS275	42
		Atherosclerosis		Overexpression of HDAC2 attenuates vascular inflammation	Arg2		59
HDAC3	Class I		VEGF	Guiding ES cells to EC differentiation	p53-p21 pathway		19
				Overexpression of HD3α reprogrammed human aortic vascular integrity	PI3K/Akt TGFβ2		44
		–	–	Inducing smooth muscle differentiation down-regulation	Notch ligand Jagged1		30
		Atherosclerosis		Increasing vascular inflammation	TGFβ1		64
		Atherosclerosis		Increasing vascular inflammation	TNF-α, IL-6, and MCP-1	RGFP966	57
		Oxygen glucose deprivation and reoxygenation		HDAC3 inhibition attenuates the increase of transendothelial cell permeability	PPARγ	RGFP966	85
		Diabetes		HDAC3 inhibition significantly reduced profibrotic cytokines PAI-1 and CTGF levels		VPA/RGFP-966	75
HDAC4	Class IIa	Hypertension		HDAC4 inhibition decreases vasoconstriction	Nitric oxide pathway	LMK235	92
		Glioblastoma		Inhibition of HDAC1/4 and phospholipase D1 decreases angiogenesis	PKCζ-Sp1 axis	SAHA	106
			Ang II	Increasing vascular inflammation	FoxO3a deacetylation		58
HDAC5	Class IIa	Hypertension		Reducing vasoconstriction and VSMC hypertrophy by HDAC5 knockdown	Ang II/ROS	TMP269/TMP195	90
HDAC6	Class IIb			Increasing microvascular endothelial cell proliferation	HDAC6/miR-155-5p/RHEB pathway		25
				HDAC6 inhibition promotes the differentiation of VSMCs	MRTF-A/SRF pathway	TSA	31
		COPD		HDAC6 inhibition blocks the cigarette smoke-induced increases in vascular remodeling	ERK pathway	TSA	101
				Combinational EGFR and HDAC2/6 inhibition reduces angiogenesis	EGFR/HDAC2/6 inhibition	3BrQuin-SAHA and 3ClQuin-SAHA	105
HDAC7	Class IIa			Enhancing the migration of ECs	PDGF-B		23
			VEGF	Enhancing EC migration and proliferation	PKC/PKD1 pathway		24
				Spliced HDAC7 isoform induces SMC differentiation from ES cells	SMA and SM22 reporter gene		29
HDAC8	Class I	Hypertension		HDAC8 inhibition decreases vasoconstriction	NO pathway	PCI34051	56
		Hypertension		HDAC8 inhibition reduces vascular inflammation	VCAM-1/ICAM-1	PCI34051	56
HDAC9	Class IIa	Atherosclerosis	–	Increasing endothelial-to-mesenchymal transition and decreasing plaque stability			68
		Atherosclerosis		Increasing vascular inflammation	IKKα/β/P65/P50	TMP195	69
		Cerebral ischemia/reperfusion injury		Inducing vascular permeability dysfunction	P62		88
HDAC10	Class IIb	Vascular restenosis	TNF-α and IGF-1	Increasing VSMC proliferation	TNF-α/IGF-1/HDAC2/10/DNMT1		39
HDAC11	Class IV	High-fat diet		Promoting EC pyroptosis	NLRP3/caspase-1/GSDMD and caspase-3/GSDME		63
SIRT1	Class III	Atherosclerosis		Repressing migration and proliferation of VSMCs	Cyclin D1/MMP-9		34
		Atherosclerosis		Preventing macrophage foam cell formation.	RelA/p65-NF-kB/oxLDL		65
		Atherosclerosis		Reducing macrophage foam cell formation	ox-LDL		70
SIRT2	Class III	Hypertension	Ang II	Stimulating microtubule redistribution and deacetylation	α-tubulin/Ang II		102
		Pathological cardiac hypertrophy		Decreasing fibrosis	LKB1/AMPK/Ang II		74
SIRT6	Class III	Hypertension		Anti-perivascular fibrosis effect	Nkx3.2-GATA5		73
SIRT7	Class III	Vascular injury		SIRT7 deficiency attenuates the VSMC proliferation	microRNA 290–295/cyclin-dependent-kinase 2		40
		Atherosclerosis		Inhibiting VSMC proliferation	Wnt/β-catenin-dependent pathway		41

Notes: AMPK, Adenosine 5‘-monophosphate (AMP)-activated protein kinase; Ang II, angiotensin II; Arg2, arginase 2; COPD, chronic obstructive pulmonary disease; CTGF, connective tissue growth factor; DNMT1, DNA methyltransferase-1; EC, endothelial cell; EGFR, epidermal growth factor; ERK, extracellular signal-regulated kinase; FoxO3a, forkhead box O3; GATA5, GATA-binding protein 5; GSDMD, gasdermin-D; GSDME, gasdermin-E; HDAC, histone deacetylase; HD3α, heading date 3α; ICAM-1, intercellular cell adhesion molecule-1; IGF-1, insulin-like growth factor 1; IL-6, interleukin- 6; IKK, inhibitor of KappaB kinase; LKB1, liver kinase B1; MCP-1, monocyte chemoattractant protein 1; MDM2, murine double minute 2; MMP-9, matrix metalloproteinase 9; MRTF-A, myocardin-related transcription factor A; MS275, Entinostat; NLRP3, NOD-like receptor thermal protein domain associated protein 3; NF-kB, nuclear factor kappa-B; Nkx3.2, NK3 homeobox 2; ox-LDL, oxidized low-density lipoprotein; PAI-1, plasminogen activator inhibitor-1; PDGF-B, platelet-derived growth factor-B; PKC, protein kinase C; PKD1, protein kinase D1; PI3K, phosphoinositide 3-kinase; PPARγ, peroxisome proliferator-activated receptor gamma; RHEB, Ras homolog enriched in brain; RNase1, ribonuclease 1; SAHA, vorinostat; SIRT, sirtuin; SM22, smooth muscle protein 22; SMA, smooth muscle actin; SMC, smooth muscle cell; sp-1, specificity protein 1; SRF, serum response factor; TGFβ1, transforming growth factor-beta 1; TGFβ2, transforming growth factor-beta 2; TNF-α, tumor necrosis factor-α; TSA, trichostatin A; VCAM-1, vascular cell adhesion molecule-1; VEGF, vascular endothelial growth factor; VPA, valproic acid; VSMC, vascular smooth muscle cell; Wnt, wingless.

## Author contributions

HJ, YXL, and MZQ designed the study. YHY, GK, and DP searched the literature and wrote the manuscript. NJY, WYY, WZY, ZYM, LGL, SCJ and PMH searched the literature and made the table, and YHY drew the figures. All authors read and approved the final manuscript.

## Conflict of interests

No potential conflict of interests was reported by the authors.

## Funding

This work was supported by the National Natural Science Foundation of China (No. 82103508, 82203758), Natural Science Foundation of Shaanxi Province (China) (No. SZY-KJCYC-2023-028), the Science and Technology Development Incubation Fund of Shaanxi Provincial People's Hospital, Shaanxi, China (No. 2021YJY-21), the Project of Tangdu Hospital, the Air Force Medical University, Shaanxi, China (No. XJSXYW202130, XJSXYW-2023015, 2021LCYJ019), the Project of Air Foce Medical University, Shaanxi, China (No. 2022LC2227), and the Talent Support Program of Shaanxi Provincial People's Hospital, Shaanxi, China (No. 2022JY-38).
